# Evaluation of surfactant removal efficiency in selected domestic wastewater treatment plants in Poland

**DOI:** 10.1007/s40201-019-00387-6

**Published:** 2019-07-10

**Authors:** Izabela Kruszelnicka, Dobrochna Ginter-Kramarczyk, Bogdan Wyrwas, Jakub Idkowiak

**Affiliations:** 1 Faculty of Civil and Environmental Engineering, Poznan University of Technology, Berdychowo 4, 60-965 Poznan, Poland; 2 Faculty of Chemistry and Technical Electrochemistry, Poznan University of Technology, Berdychowo 4, 60-965 Poznan, Poland; 3 Institute of Bioorganic Chemistry Polish Academy of Sciences, Z. Noskowskiego 12/14, 61-704 Poznan, Poland

**Keywords:** Sewage-treatment plant, Sand filter, Domestic sewage, Surfactants, Efficiency

## Abstract

The aim of this study was to evaluate the work of a two types of household sewage treatment plant: wetland wastewater treatment plant (ORS type) and treatment plant of SBR type (SBR-K-6 type). Physicochemical analyses of selected pollution indices (BOD_5_, COD, total suspension, total phosphorus) and surfactants were carried out and compared with currently applicable values of such indexes according to the Regulation of the Minister of the Environment in Poland on the conditions to be met when discharging sewage into water or soil, and on the substances particularly harmful to the aquatic environment. The removal efficiency of organic compounds, expressed as COD and BOD_5_, reached the threshold of 90%, which is required in regulations. In contrast, the effects of removal of biogenic compounds were low – in case of total nitrogen the removal rate reached approx. 40% and the desired admissible concentration of 30 mg N/L was not achieved. The reduction efficiency of total suspended solids reached 57.0 and 59.6% for the ORS and SBR-K-6 type objects, respectively, and therefore the required threshold of minimum 90% was not reached. Anionic surfactants were removed by up to 98 and 88% in the ORS and SBR-K-6 type wastewater treatment plants, respectively. Lower removal efficiency was achieved in case on non-ionic surfactants, which reached 76% for the ORS type object and 56% for the SBR-K-6 type object. This article proven high wastewater treatment efficiency and lower than necessary concentrations in the effluent from domestic wastewater treatment plants may be achieved mainly by proper exploitation of the devices and appropriately selected vegetation.

## Introduction

To date, the wastewater management is not adequately regulated in rural areas. Due to considerable distances between buildings and frequently occurring inconvenient conditions for building a collective sewage, the methods for treatment of sewage in single buildings have become widely propagated in Regulation of the Polish Minister of Environment of 18 November 2014 on the conditions to bemet when introducing sewage into water or soil and on substances particularly harmful to the aquatic environment [1, 2]. Such solutions should be characterized by high operational efficiency, reliability and cost-effectiveness. “Mini” wastewater treatment plants with activated sludge are commonly used for treatment of low-volume wastewater streams, which are a miniaturized version of technologies used in big objects. In case of residential wastewater treatment plants (RWTPs) which work with the use of the classic activated sludge, their proper operation is negatively affected by shifts in the pollutant load and wastewater volume as well as changing environmental conditions. The highest threat for such systems is associated with the presence of surface active compounds (surfactants) [3–7]. In 2010 the production of surface active agents in Poland reached 81 thousand tons. After 6 years it was doubled and currently it exceeded the level of 165 thousand tons as reported Small Statistical Yearbook of Poland [8].

The increasing demand for different types of surface active products in numerous branches of everyday life, which contributes to their increasing production, resulted in the fact that currently this is the main source of synthetic organic carbon introduced into surface water.

Surfactants and products of their incomplete biodegradation continually enter wastewater and ground water streams. The contribution of surface active compounds, especially the group of non-ionic surfactants, is progressively increasing, therefore their control and searching for efficient means of limiting this type of contamination is of importance. Surfactants enter the environment due to incorrectly operating RWTPs. Applied at high amounts these compounds are the main source of organic carbon. They enter the aqueous environment as detergents in the form of washing agents, cleaners, emulgators and additives [9]. Their detergent properties, i.e. their ability to remove stains from the surface of solids and keep them dispersed in the washing solution, contribute to their broad spectrum of applications. These properties may be exhibited by a single substance, however it is more efficient to use mixtures of chemical compounds. Anionic and non-ionic surfactants are the main components of detergents, which display a tendency to gather at the interfacial boundary (e.g. solid/liquid) due to the fact that they comprise both hydrophilic and hydrophobic groups in their structure. Their adsorption properties enable the wetting of substances as well as the removal and dispersion of dirt particles.

Recently, a tendency to use washing and cleaning agents in concentrated forms can be observed. However numerous users do not follow the producers guidelines which results in an overdose. The consequence of such actions is an increased amount of surfactants carried with wastewater. Cationic surfactants display the highest toxicity, however it is difficult to evaluate their ecological hazard, since their share in the global production of surface active compounds doesn’t exceed 7–10% [10]. Furthermore, they form complexes with anionic surfactants which are present at much higher concentrations in wastewater [11]. It was established that such complexes are characterized by several times lower toxicity compared to pure cationic compounds. Moreover, if the summary amount of carbon atoms in the complex exceeds 22 atoms, the compound will precipitate. Anionic surfactants are approx. 3 times less toxic compared to cationic surfactants, however, due to the wide scale of their production and application, their presence in the environment is a serious issue. The negative effects associated with their presence in the aqueous environment includes [12, 13]:

- problems with oxygen diffusion into aqueous systems,
- foaming,
- increased solubility of pesticides and other plant protection agents,
- disruption of auto-remediation processes in aqueous systems,
- toxicity towards aquatic organisms.

Surfactants also inhibit the biodegradation of organic compounds and the denitrification of ammonium nitrogen. They have a negative influence on the structure of flocks in the activated sludge. It is estimated that changes of flock structure occur at a weight concentration of 0.01% of surfactants. Higher concentrations result in cell lysis [14].

This article presents the research results focused on evaluating the operational efficiency of a residential wastewater treatment plant in terms of removing pollutants, especially anionic and non-ionic surfactants. Furthermore, the methods for determination of surfactants were also elucidated. Due to the fact that wastewater may contain several thousands of compounds and are characterized by constantly shifting qualitative and quantitative composition, traditional instrumental methods are inadequate for a proper analysis. Despite the high diversity of the annually published methods dedicated to the determination of surface active compounds, it is difficult to indicate simple, cost-efficient and accurate methods which would be useful for routine monitoring of surface water and wastewater, especially with regard to non-ionic surfactants [15–17]. The chemical structure of oxyethylates hinders the development of methods appropriate for their trace analysis. Several compounds belonging to this group do not comprise strong chromophore groups, which prevents their determination using UV-Vis spectrophotometry or detection using chromatography in flow injection systems.

Standard determination methods of surface active compounds with high limit of detection, low precision and high labour intensity often do not comprise with the standards of novel environmental analysis [18].

The use of modern instrumental techniques for the analysis of surface active compounds is notably limited by the complexity of the analyte. Surfactants are a mixture of homologues and their complex chromatograms as well as NMR *(Nuclear magnetic resonance spectroscopy)* and mass spectra can rarely be interpreted in a precise manner. An additional issue is associated with a complex matrix of highly contaminated environmental samples (raw and treated wastewater, surface water). Commercial test cuvettes, although rapid and easy to use, are not entirely suitable for environmental, monitoring of surface active compounds due to low sensitivity and high susceptibility to the influence of contaminants present in the matrix [19–24].

The multi-dimensional evaluation of operational efficiency of residential wastewater treatment plant was conducted in order to compare the contaminant removal rate guaranteed by the producer – which complies with the Regulation of the Minister of the Environment on the conditions to be met when discharging sewage into water or soil, and on the substances particularly harmful to the aquatic environment [1] – and the actual physical state resulting from everyday exploitation of devices in average households.

## Materials and methods

### Experimental

**Fig. 1:**
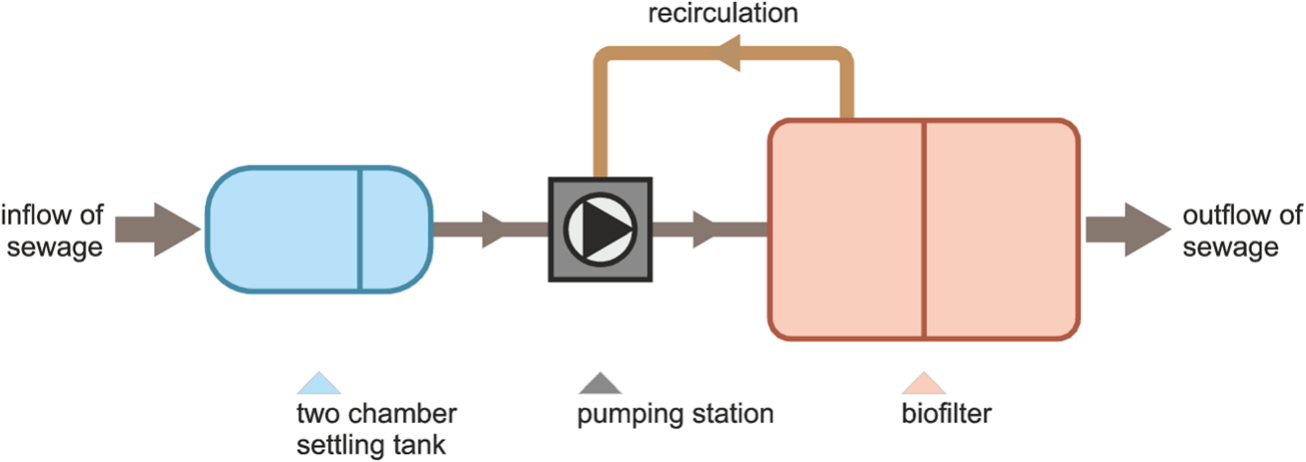
Scheme of the ORS type unit

**Fig. 2:**
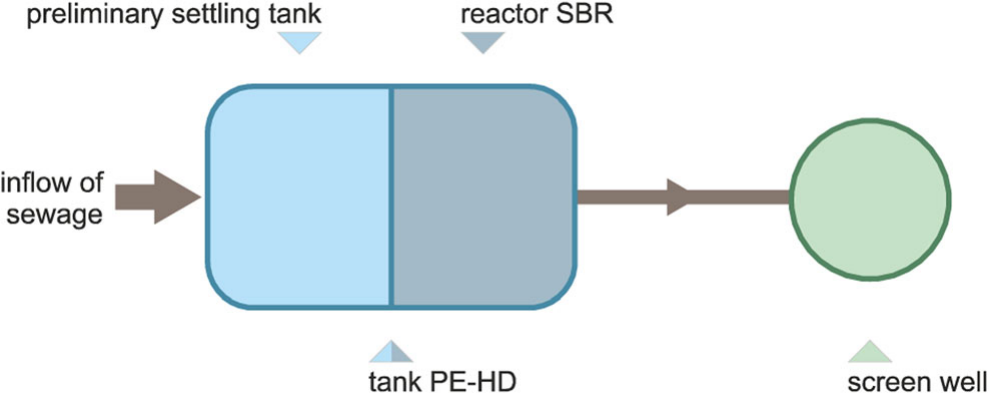
Scheme of the SBR-K-6 type unit

Samples were collected from two different objects localized in the central part of the Wielkopolska region, in the Poznan district. Two selected individual wastewater treatment systems are presented, such as constructed wetland wastewater treatment plant with recirculation and connected with stabilization pond (ORS type) (Fig. 1) and treatment plant of SBR (*sequencing batch reactor)* type (SBR-K-6 type) (Fig. 2).

In wetland treatment plants a complex system is responsible for the treatment process, which includes: water, mineral base, parts of dead plants, living plants and wide variety of microorganisms (bacteria, protozoa, fungi) as well as other organisms (invertebrates and vertebrates). Such diversity of organisms in the treatment system contributes to numerous mechanism which allow to remove contaminants in wastewater. The studied wastewater treatment plant was designed with an assumption that the daily unit water consumption will be at 130 L per day per person. The number of users supported by the object for 4 persons. The technological parameters of the tested object

- the total volume 3,4 m^3^,
- the volume of the preliminary settling tank 2 m^3^
- the bed surface – 21 m^2^
- the kind of plants – *Miscanthus sinensis Gracimillus, Miscanthus sinensis Littre Silver Spider, Miscanthus sinensis Morningh Light, Miscanthus sinensis Nippon, Miscanthus sinensis Zebrinus, Miscanthus sinensis David, Pennisetum alopecuroides Hameln, Pennisetum alopecuroides Viridiscens, Carex buchannanii Red Rooster, Carex comans Frosted curls.*

Method of calculation of the wastewater amount for the ORS-type treatment plant presented in Table 1.

**Table 1:** Method of calculation of the wastewater amount for the ORS-type treatment plant

Formulas for calculation		Result	
Q_d, avg_ = 0.13 · LM	*[m*^*3*^*]*	Q_d, avg_ = 0.13 · 4 = 0.52	*m*^*3*^
Q_d, max_ = N_d, max_ · Q_d, avg_	*[m*^*3*^*/d]*	Q_d, max_ = 2.5 · 0.52 = 1.3	*m*^*3*^*/d*
${\mathrm{Q}}_{\mathrm{h},\max }={\mathrm{Q}}_{\mathrm{d},\max}\cdotp \frac{{\mathrm{N}}_{\mathrm{h},\max }}{24}=\frac{{\mathrm{Q}}_{\mathrm{d},\max }}{6}$	*[m*^*3*^*/h]*	${\mathrm{Q}}_{\mathrm{h},\max }=\frac{1.3}{6}=0.217$	*m*^*3*^*/h*
Q_year_ = Q_d, avg_ · 365	*[m*^*3*^*/year]*	Q_year_ = 0.52 · 365 = 190	*m*^*3*^*/year*

The second studied object was a SBR (*Sequencing batch reactor*) K 6 type domestic wastewater treatment plant (Fig. 2). The SBR technology is based on sequential reactors, in which the treatment process occurs periodically. The first chamber plays the role of a preliminary settling and buffering tank, which allows for the initial mechanical treatment of wastewater by sedimentation and balance of loads caused by uneven supply of wastewater. Upon preliminary treatment, the wastewater enter the SBR chamber, in which they are aerated and purified. The aeration supplies the activated sludge microorganisms with oxygen in order to increase the treatment efficiency. The final step of treatment is the discharge of purified wastewater and recirculation of activated sludge. SBR treatment plant works in treatment cycles. One cycle may be divided into several phases. The treatment cycle lasts 7 to 8 h depending on the settings. The studied wastewater treatment plant was designed with an assumption that the daily unit water consumption will be at 130 L per day, per person. The number of users supported by the object for 6 persons. The technological parameters of the tested object

- the total volume 3,.4 m^3^,
- the volume of the preliminary settling tank 1,.2 m^3^
- the volume of reactor 2,.2 m^3^
- nominal flow 0,.9 m^3^ per day.

In both cases the physicochemical analyses of selected contaminant indicators (BOD_5_, COD, total suspended solids, total phosphorous as well as anionic and non-ionic surfactants) were conducted and compared with the current admissible concentrations in wastewater, included in the Regulation of the Minister of the Environment in Poland [1]. Samples were taken during the period from 25 April to 30 May 2016 years at a frequency of once a week.

### Determination of surface active compounds

*Modified Dragendorff reagent* (iodine bismuth(III) complex in the presence of barium ions with a reducer).

A 1.2 g dose of bismuth(III) nitrate, 150 mL of glacial acetic acid, 100 g of barium chloride, 50 g of potassium iodine 5 g of anhydrous sodium phosphate (NaH_2_PO_2_) were introduced into a 1000 mL volumetric flask. The flask was supplemented with demineralized water to a volume of 1 L and the content was mixed used a magnetic stirrer until complete dissolution of components was achieved. The obtained light orange solution was filtered using a paper filter, Whatman® qualitative filter paper, Grade 1 (Whatman Article No., 28,.413,.923). The obtained Dragendorff reagent with a reducer is characterized by rapid action and remains effective even after several years of storage in contrast to the classic reagent (without the reducer) which allows the precipitation of surfactants only within **2** weeks in accordance with the procedure described in Determination of anionic surfactants by measurement of MBAS methylene blue index PN-EN 903:2002 **[**25**]**.

*Dissolving-complexing reagent* (15% solution of thiourea in 1 M HNO_3_). A flask (100 mL volume) was filled with 60 mL of water and 6.9 mL of concentrated 65% HNO_3_. Next, 15 g of thiourea was added and the flask was supplemented with demineralized water. Crystals of thiourea were dissolved using a magnetic stirrer.

### Apparatus

#### Spectrophotometer

V-530 UV-Vis spectrophotometer (Jasco, Japan) with the ability to acquire spectra in the range of 200–1100 nm was used order to determine the content. The spectra were recorded using a standard PC. Glass cuvettes were used in the studies (10 mm × 10 mm × 30 mm) with an 10 mm optical path length. The absorbance for wavelength λmax = 650 nm was used as the analytical signal for determination of anionic surfactants, whereas the wavelength λmax = 468 nm was used in case of non-ionic surfactants.

#### MBAS method for determination of anionic surfactants

Deminarelized water (100 mL), an appropriate amount of the sample including anionic surfactants, 10 mL of carbonate buffer (pH = 10) and 5 mL of neutral methylene blue solution were introduced into a 250 mL separatory funnel. Next, chloroform was added in a stepwise manner using doses of 15, 10 and 10 mL. After each dose the sample was shaken for 3 min and introduced to a second separatory funnel, which contained 110 mL of demineralized water and 5 mL of acidic methylene blue solution. After the shaking step, the chloroform phase from the first separatory funnel was introduced to a second separatory funnel, then shaken again in order to purify the blue chloroform extract. The extracts from the second separatory funnel were introduced to a volumetric flask (50 mL) and supplemented with chloroform to a final volume of 50 mL. The prepared solution was filtered using a paper filter to a glass cuvette and adsorption spectra were measured against chloroform at λmax = 650 nm what it assumes Regulation of the Minister of Environment No. 1658 on reference methodologies for testing the degree of biodegradation of surfactants contained in products whose use may affect water quality [26].

**Fig. 3:**
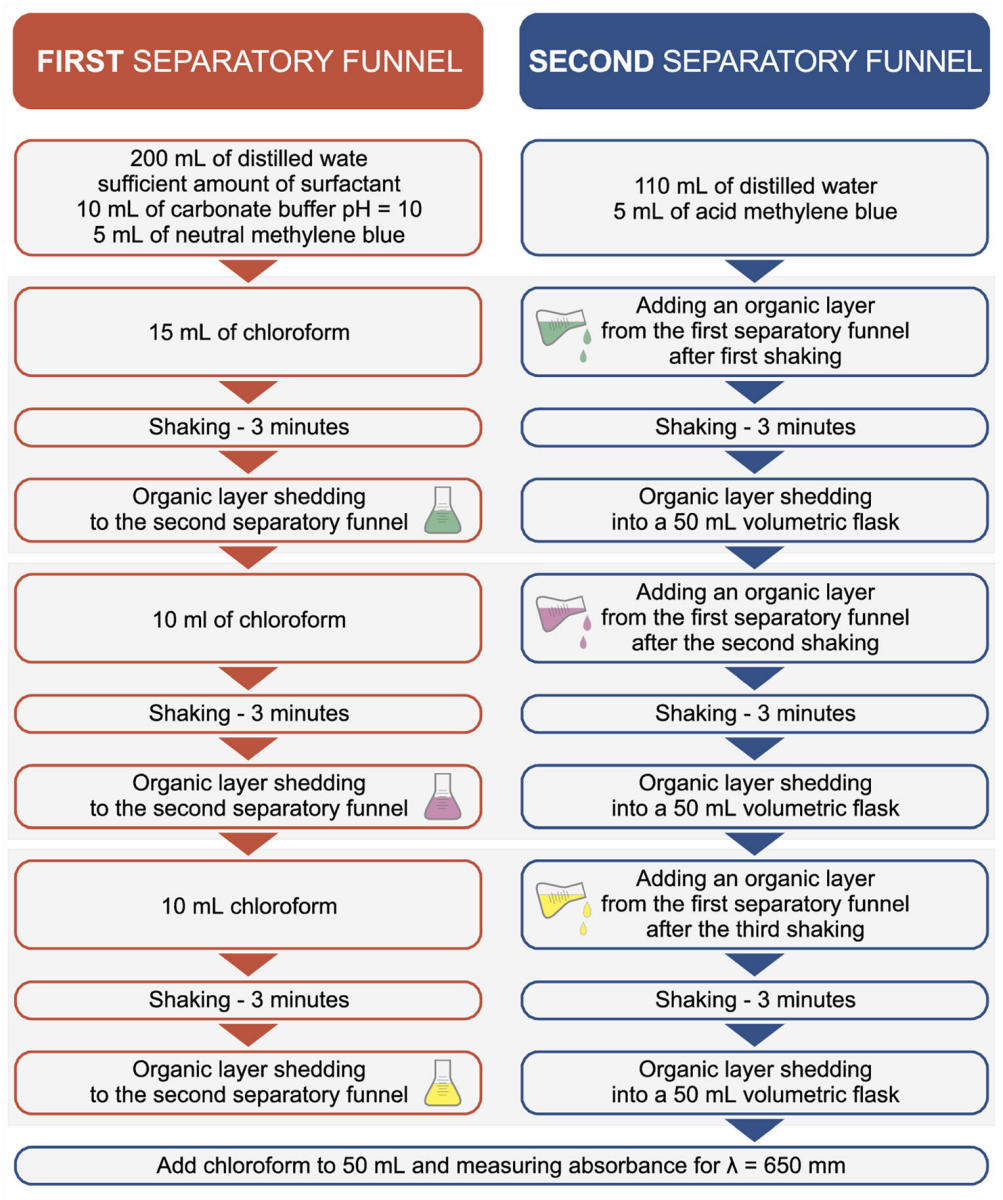
Scheme of the MBAS procedure for determination of anionic surfactants

The mean result was obtained using 3 independent measurements for each sample. The relative standard deviation of the method is at 7.5%. Additionally, the correctness of the employed procedure was controlled by addition of a standard. The results of the measurements of anionic surfactants in the wastewater samples were investigated in relation to an anionic surfactant, sodium dodecylbenzenesulfonate. A complex scheme of the MBAS procedure is presented in Fig. 3.

#### Modified BiAS-thio method for determination of non-ionic surfactants

**Fig. 4:**
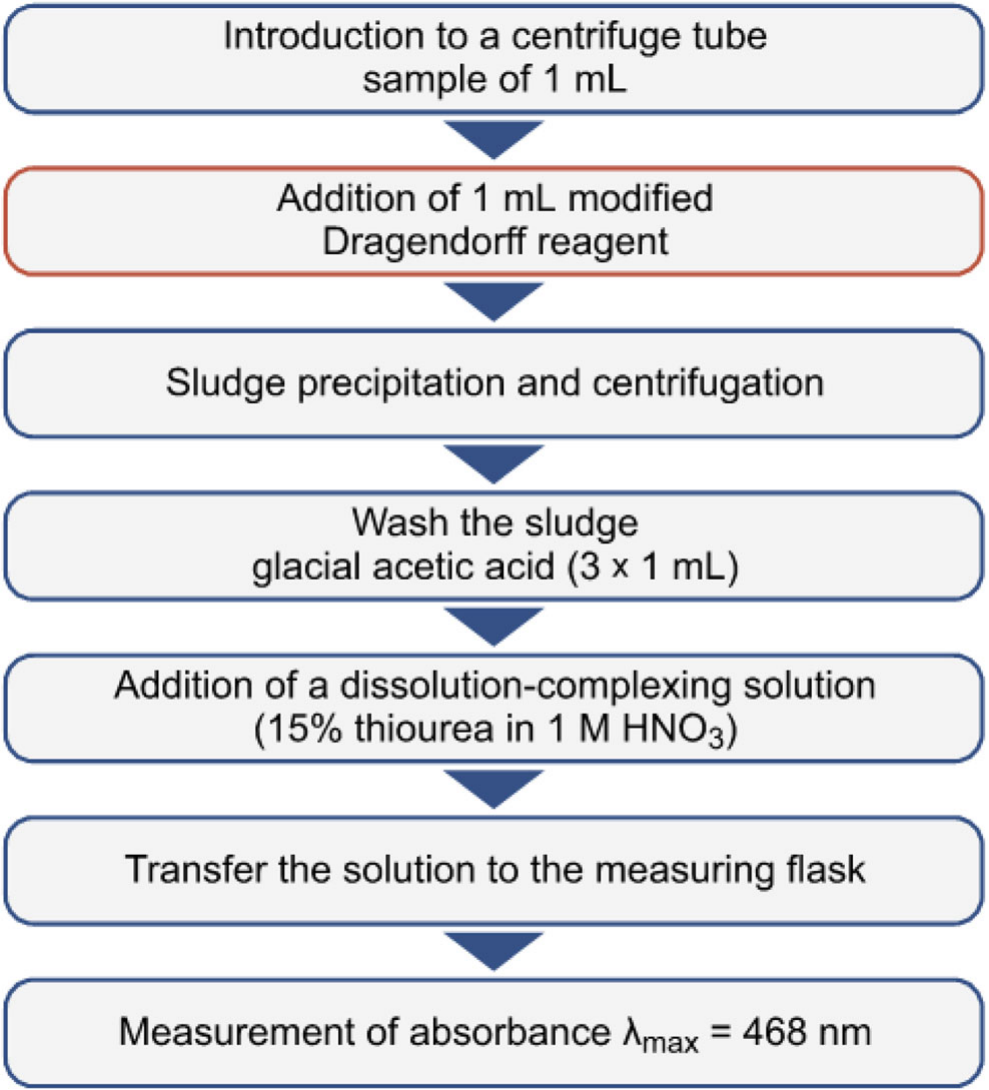
Scheme of the BiAS-tio procedure for determination of non-ionic surfactants

Approx. 1 mL of the solution including non-ionic surfactants and 1 mL of the modified Dragendorff reagent were introduced into centrifuge tubes. Next, the tubes were centrifuged (16,000 rpm for 5 min.). After precipitation and separation of the oxyethylate precipitate using the modified Dragendorff reagent, the solution was decanted and the orange precipitate was rinsed 3 times using 1 mL of glacial acetic acid. In order to remove the residual Dragendorff reagent. The precipitate was dissolved in 2 mL of the dissolving-complexing solution (15% solution of thiourea in 1 M HNO_3_) and placed in 1 cm glass cuvette. Absorbance was measured for λ max = 468 nm against demineralized water. The relative standard deviation of the method is equal to 6.6% [27]. The mean result was obtained using 3 independent measurements for each sample. Additionally, the correctness of the employed procedure was controlled by addition of a standard. A scheme of the modified BiAS-tio procedure is presented in Fig. 4. The results were calculated using a model non-ionic surfactant – Triton X-100.

## Results and discussion

On the basis of conducted studies it was established that both ORS and SBR-K-6 type wastewater treatment plants comply with the requirements regarding chemical and biochemical oxygen demand. The average COD concentration for raw sewage at the inflow to the ORS treatment plant was 815.2 mg O_2_/L, and for the SBR-K 6, 759.8 mg O_2_/L The average concentration of COD in treated wastewater was 37.4 mgO_2_/L in the case of the ORS treatment plant and 82.3 mgO_2_/L in the case of the SBR-K 6 treatment plant. In the case of BOD_5_, the average concentration for raw sewage at the inflow to the ORS treatment plant was 353.6 mgO_2_/L, and for the SBR-K 6, 504.3 mgO_2_/L. The average BOD_5_ concentration in treated wastewater was 7.3 mgO_2_/L for the ORS treatment plant and 28.0 mgO_2_/L for the SBR-K 6.

The mean reduction of COD in the case of the ORS sewage treatment plant was 95.0%, while the BOD5 reduction was 97.1%. In the case of the SBR-K-6 sewage treatment plant, the reduction of these indicators reaches 89.5% and 93.6%. According to regulation [1], the maximum permissible COD value for domestic or municipal sewage introduced into the aquatic or terrestrial environment is 150 mg O_2_/L. For BOD_5_, these values ​​are 25 mgO_2_/L *or minimal* pollutant *reduction 70–90%* for purified *wastewater introduced into* the aquatic environmental or at least 20% if it is introduced into the ground [1]. The reduction of BOD_5_ and COD above 90% was obtained as a result of purification in sewage treatment plants of the ORS and SBR-K-6 type.

The reduction of anionic and non-ionic surfactants content was also investigated in the studied domestic wastewater treatment plants. Considering the content of anionic and non-ionic surfactants in the wastewater effluents which exited the wastewater treatment plants, it can be established that surfactants were removed more efficiently in the ORS type plant. On the average, the results obtained for anionic surfactants determined using the MBAS method reached 0.23 mg/L for the ORS type object and 0.81 mg/L for the SBR-K-6 type object. Similar results were obtained in case of non-ionic surfactants determined using the BiAS-tio method. The mean content of non-ionic surfactants reached 2.53 mg/L for the ORS type object and 6.45 mg/L for the SBR-K-6 type object. Upon analysis of reduction rates for anionic surfactants (Table 2), it was established that both objects were characterized by excellent treatment efficiency of such contaminants. The mean reduction rate for the ORS type object reached 98.3%, whereas in case of the SBR-K-6 object the value was at 88.2%.

**Table 2:** Reduction rate of anionic surfactants (MBAS method)

Sample number	ORS *degree of reduction [%]*	SBR-K-6 *degree of reduction [%]*
III	98.9	96.0
IV	97.2	74.5
V	98.7	94.2
average	98.3	88.2

The biodegradation process was less efficient in case of non-ionic surfactants. On the average, the reduction rate reached 76.1% for the ORS type wastewater treatment plant and 56.2% for the SBR-K-6 type wastewater treatment plant (Table 3).

**Table 3:** Reduction rate of non-ionic surfactants (BiAS-tio method)

Sample number	ORS *degree of reduction [%]*	SBR-K-6 *degree of reduction [%]*
III	100.0	81.3
IV	28.2	30.2
V	100.0	57.1
average	76.1	56.2

The conducted studies regarding the reduction rate of surfactants indicated that their removal efficiency is relatively high. It may be observed that the reduction of anionic surfactants proceeded more efficiently and exceeded 80%. The removal rate of non-ionic surfactants is lower, at approx. 60%. In comparison, it is worth noting that the removal rate of anionic surfactants in a big and fully operational municipal wastewater treatment plan (e.g. The Central Wastewater Treatment Plant for Poznan city) is at approx. 90%, while in case of non-ionic surfactants it reaches 80%. The legal regulations regarding the amount of surface active compounds are addressed in the Regulation of the Minister of Construction from the 14th of July 2006 regarding the method of fulfilling the obligations of industrial wastewater providers and conditions for introducing the wastewater into sewage facilities and determine the admissible concentrations for the remaining pollution indices in industrial wastewater introduced into sewage facilities: anionic surfactants - 15 mg/L, non-ionic surfactants - 20 mg/L [28].

The results of determination of total nitrogen content are presented in Table 4. The mean nitrogen concentration in the raw wastewater was at 78.50 mgN/L for the ORS type object and 60.50 mgN/L for the SBR-K-6 type object. After the treatment process the mean concentration of nitrogen in the effluent from the ORS type wastewater treatment plant reached 42.86 mgN/L whereas in case of the SBR-K-6 type wastewater treatment plant the value was at 34.35 mg N/L. Mean reduction rate was at 45.7 and 37.3% for the ORS and SBR-K-6 type objects, respectively. According to regulation [1], in both cases nitrogen reduction allows the introduction of treated wastewater to the ground. However, in the case of introducing sewage treated to lakes and flowing waters, this value is exceeded. The maximum permissible total value of nitrogen for domestic sewage introduced into the aquatic environment is 30 mgN/L.

**Table 4:** Results of determination of total nitrogen content

Sample number	ORS *[mg/L]*		SBR-K-6 *[mg/L]*	
	inflow	outflow	inflow	outflow
I	59.0	22.2	–	–
II	102.0	51.0	60.2	46.5
III	76.0	58.5	65.4	30.3
IV	81.5	37.0	34.1	31.1
V	74.0	45.6	82.3	29.5
average	78.5	42.9	60.5	34.4

After the treatment process the mean total suspended solids content (Table 5) in the effluent from the ORS and SBR-K-6 type wastewater treatment plants was at 82.4 and 99.3 mg/L, respectively. According to the regulation [1], the maximum permissible total suspended solids content in sewage introduced into the aquatic environment (lakes and flowing water) is 35 mg/L or 90%, while in the case of introducing sewage into the ground, the reduction rate must be below 50%. The average reduction of the total suspension in the investigated cases was 57.0% and 59.6% for ORS and SBR-K-6, respectively.

**Table 5:** Results of determination of total suspension

Sample number	ORS *[mg/L]*		SBR-K-6 *[mg/L]*	
	inflow	outflow	inflow	outflow
I	424.0	96.0	–	–
II	121.0	76.0	237.0	113.0
III	396.0	64.0	308.0	116.0
IV	24.0	136.0	504.0	52.0
V	80.0	40.0	176.0	116.0
average	155.3	82.4	306.3	99.3

## Conclusions

The obtained data and conducted analyses indicated that the wastewater treatment processes realized in domestic wastewater treatment plants allows for efficient reduction of pollutants in wastewater. The removal efficiency of organic compounds, expressed as COD and BOD_5_. In contrast, the effects of removal of biogenic compounds were low – in case of total nitrogen the removal rate reached approx. 40% and the desired admissible concentration of 30 mg N/L was not achieved. The reduction efficiency of total suspended solids reached 57.0 and 59.6% for the ORS and SBR-K-6 type objects, respectively, and therefore the required threshold of minimum 90% was not reached. The data regarding the removal of surface active compounds were very promising. Anionic surfactants were removed by up to 98 and 88% in the ORS and SBR-K-6 type wastewater treatment plants, respectively. Lower removal efficiency was achieved in case on non-ionic surfactants, which reached 76% for the ORS type object and 56% for the SBR-K-6 type object.

High wastewater treatment efficiency and lower than necessary concentrations in the effluent from domestic wastewater treatment plants may be achieved mainly by proper exploitation of the devices and appropriately selected vegetation, which may considerably improve the reduction rate of nitrogen and phosphorous in the wastewater by accumulation of biogenic compounds.
